# Reduced erythrocytic CHCHD2 mRNA is associated with brain pathology of Parkinson’s disease

**DOI:** 10.1186/s40478-021-01133-6

**Published:** 2021-03-08

**Authors:** Xiaodan Liu, Qilong Wang, Ying Yang, Tessandra Stewart, Min Shi, David Soltys, Genliang Liu, Eric Thorland, Eugene M. Cilento, Yiran Hou, Zongran Liu, Tao Feng, Jing Zhang

**Affiliations:** 1 Department of Pathology, School of Basic Medical Sciences, Peking University Third Hospital, Peking University Health Science Center, Beijing, 100191 China; 2 Beijing Municipal Key Laboratory of Biomarker and Translational Research in Neurodegenerative Diseases, Beijing, China; 3 Department of Pathology, School of Medicine, University of Washington, Seattle, WA USA; 4 Center for Movement Disorders, Department of Neurology, Beijing Tiantan Hospital, Capital Medical University, Beijing, 100070 China; 5 China National Clinical Research Center for Neurological Diseases, Beijing, 100070 China; 6 The Affiliated High School of Peking University, Beijing, 100080 China; 7 Parkinson’s Disease Center, Beijing Institute for Brain Disorders, Capital Medical University, Beijing, 100069 China; 8 Department of Pathology, The First Affiliated Hospital and School of Medicine, Zhejiang University, Hangzhou, China; 9 China National Health and Disease Human Brain Tissue Resource Center, Hangzhou, China

**Keywords:** Parkinson’s disease, Mitochondria dysfunction, α-synuclein, CHCHD2

## Abstract

Peripheral biomarkers indicative of brain pathology are critically needed for early detection of Parkinson’s disease (PD). In this study, using NanoString and digital PCR technologies, we began by screening for alterations in genes associated with PD or atypical Parkinsonism in erythrocytes of PD patients, in which PD-related changes have been reported, and which contain ~ 99% of blood α-synuclein. Erythrocytic CHCHD2 mRNA was significantly reduced even at the early stages of the disease. A significant reduction in protein and/or mRNA expression of CHCHD2 was confirmed in PD brains collected at autopsy as well as in the brains of a PD animal model overexpressing α-synuclein, in addition to seeing a reduction of CHCHD2 in erythrocytes of the same animals. Overexpression of α-synuclein in cellular models of PD also resulted in reduced CHCHD2, via mechanisms likely involving altered subcellular localization of p300 histone acetyltransferase. Finally, the utility of reduced CHCHD2 mRNA as a biomarker for detecting PD, including early-stage PD, was validated in a larger cohort of 205 PD patients and 135 normal controls, with a receiver operating characteristic analysis demonstrating > 80% sensitivity and specificity.

**Supplementary Information:**

The online version contains supplementary material available at 10.1186/s40478-021-01133-6.

## Introduction

Diagnosis of Parkinson’s disease (PD), a common neurodegenerative disorder [28], is currently based on a combination of medical history, observation of cardinal motor indicators, and response to pharmaceutical therapies [42, 47]. However, motor symptoms often present after greater than 50% of affected neurons have degenerated [4]. Further, a more definitive diagnosis cannot be made until a sustained therapeutic response is observed during additional years of follow-up. In other words, there is an essential need to develop a method for accurate diagnosis of PD at early stage, where disease modifying therapies are likely to be most effective.

Much effort in the PD biomarker field has focused on the discovery of biomarkers using the cerebrospinal fluid (CSF), with α-synuclein and its variants receiving most attention [32, 41, 45]. However, very few markers investigated thus far demonstrate clinically useful power in detecting PD or following PD progression, and none has been widely validated. Alongside the challenges associated with these protein markers, CSF collection (via lumbar puncture), often perceived as a high-risk and painful procedure, is impractical for routine screening purposes. More recently, investigations on PD-related proteins in blood have received more consideration; however, they have not yielded consistent results, largely because of the complexity of the samples [24]. In contrast, blood-based changes in mRNA expression present an additional and promising biomarker strategy for differentiating PD patients from healthy controls [3, 31, 36]. One study compared the transcriptional profile of PD patients with or without postural instability and found > 200 differentially expressed genes, some of which were also found to be dysregulated in a dopaminergic cell model of PD [26], suggesting that altered gene expression in the blood may reflect changes in the central nervous system (CNS). Another group explored the genetic signature in blood from a large cohort of PD patients and identified 87 genes which differentiated between patients with idiopathic PD and controls [36], further supporting the concept of altered gene expression within the blood as a useful tool for predicting PD. Notably, both studies used RNA extracted from whole blood cells, mainly leukocytes, which undergo nuclear and transcriptional changes during disease states. Compared to leukocytes (and most other blood cells), mature erythrocytes are uniquely structured, lacking organelles and nuclei necessary for replacing dysfunctional proteins. Thus, alterations in residual RNA and protein levels in erythrocytes likely reflect pathological rather than physiological changes [2]. Additionally, ~ 99% of blood α-synuclein is located in erythrocytes, and pathological changes in erythrocytes have been described in PD patients in several independent investigations [1, 2, 14, 21, 23, 30, 40, 44]. Therefore, in this study, we tested the hypothesis that erythrocytes carry biomarkers that reflect or correlate with brain pathology, and are capable of detecting PD at early stages.

## Results

### Reduced mRNA and protein expression of CHCHD2 in erythrocytes of PD patients

**Fig. 1:**
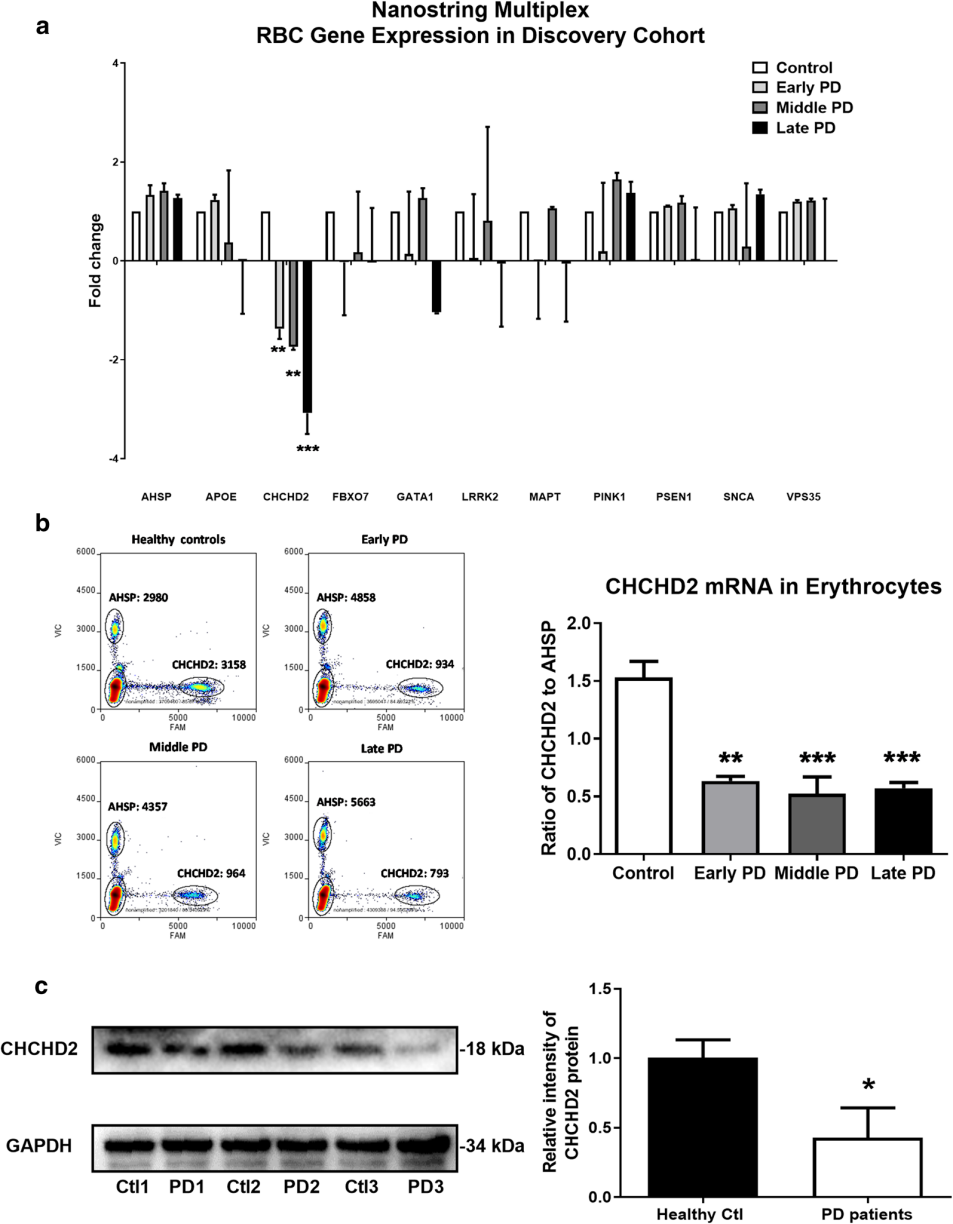
CHCHD2 mRNA and protein expression was reduced in erythrocytes of PD patients. **a** Genes associated with PD or atypical Parkinsonism in pooled erythrocyte samples from controls and PD patients at various stages were analyzed by the NanoString multiplex gene expression method. The samples were pooled according to Additional file 1: Table S1 [four comparison groups (n = 12 each) with each pooled into three sub-groups]. Ten genes were analyzed following removal of low expression genes. Significantly decreased CHCHD2 mRNA was detected in all PD groups compared to the control group [F (3, 4) = 52.0, one-way ANOVA, n = 3; *p* < 0.01 for Early or Mid PD vs. control, *p* < 0.001 for Late PD vs. control]. **b** Validation of the discovery cohort by ddPCR in the discovery set also identified decreased CHCHD2 mRNA in erythrocytes of all PD groups [F(3, 8) = 20.80, one-way ANOVA, n = 3; *p* < 0.01 or 0.001 for each PD group vs. the controls] compared to controls. No difference in CHCHD2 mRNA expression was observed between PD groups by Tukey’s multiple comparisons test. Left: Original representative ddPCR picture. FAM channel (Axis X) indicates CHCHD2, VIC channel (Axis Y) indicates AHSP. Right: Statistical result. **c **Decreased CHCHD2 protein in erythrocytes of PD patients. Proteins were extracted from pooled erythrocytes of the same cohort as used in the NanoString and ddPCR studies. Compared to controls, CHCHD2 was decreased significantly from 1.01 ± 0.07 to 0.43 ± 0.12 (*p* < 0.05, Mann–Whitney U test, n = 3)

A NanoString multiplex gene expression method was used to screen for potential mRNA biomarkers for PD within erythrocytes. A panel of 21 genes associated with PD or atypical Parkinsonism (see “Materials and methods” section for a complete list) chosen based on previous reports [6, 18, 20, 34, 43], was examined in a cohort consisting of 48 participants separated according to four diagnostic classifications: control, early-stage PD (Early PD), middle-stage PD (Mid PD), or late-stage PD (Late PD) patients according to Unified Parkinson’s Disease Rating Scale (UPDRS) score (Additional file 1: Table S1). mRNA expression frequency of each gene in erythrocytes of healthy control is shown in Additional file 1: Fig. S1, with SNCA, FBXO7, CHCHD2, PSEN1, LRRK2, VPS35, GATA1, MAPT, APOE, and PINK1 among the ten most highly expressed genes in erythrocytes. Of these, only CHCHD2 mRNA consistently demonstrated significantly reduced expression in all PD groups compared to the control group [Fig. 1a, F(3, 4) = 52.0, one-way ANOVA, n = 3; *p* < 0.01 for Early or Mid PD vs. control, *p* < 0.001 for Late PD vs. control]. This result was next confirmed by digital droplet PCR (ddPCR), which also detected significantly reduced CHCHD2 mRNA in erythrocytes across all PD groups compared to controls [Fig. 1b, F(3, 8) = 20.80, one-way ANOVA, n = 3; *p* < 0.01 or 0.001 for each PD group vs. the controls]. There were no differences, however, between PD groups with different disease severity. Having validated the mRNA result independently, we next examined the protein expression of CHCHD2 in erythrocytes of PD patients. As shown in Fig. 1c, the relative CHCHD2 protein levels were decreased significantly from 1.01 ± 0.07 in pooled erythrocytes of controls to 0.43 ± 0.12 in those of PD patients (*p* < 0.05, Mann–Whitney U test, n = 3).

### Reduced expression of CHCHD2 in the post-mortem *substantia nigra* slices of PD patients

**Fig. 2:**
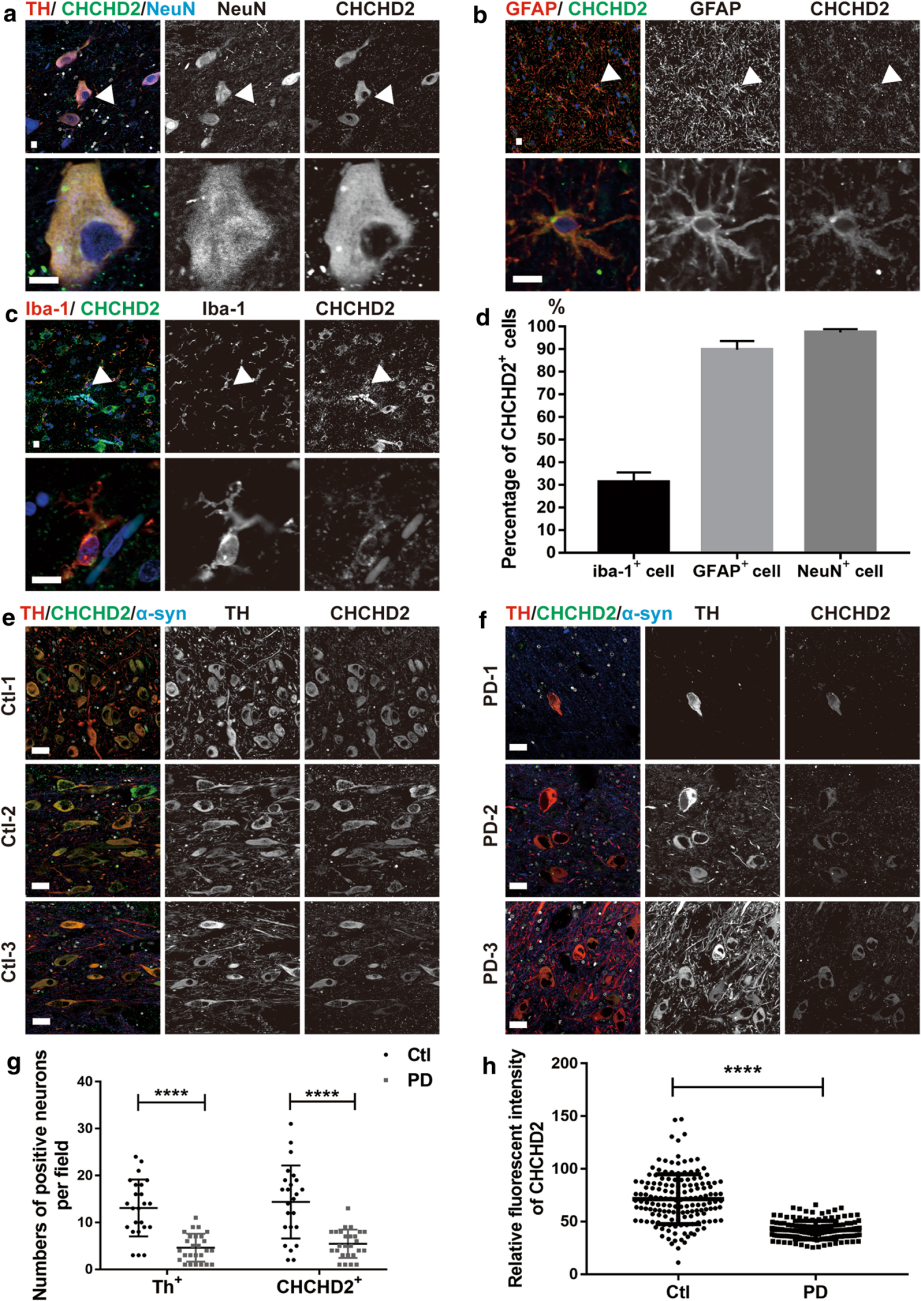
Reduced expression of CHCHD2 in post-mortem *substantia nigra* slices of PD patients. **a** Representative images of co-staining of CHCHD2 (green) with neuronal marker NeuN (blue) and dopaminergic neuronal marker TH (red), showing expression of CHCHD2 in neurons. Scale bar: 10 μm. **b** Representative images of co-staining of CHCHD2 (green) with astrocyte marker GFAP (red), showing expression of CHCHD2 in astrocytes. Scale bar: 10 μm. **c** Representative images of co-staining of CHCHD2 (green) with microglial marker Iba1 (red), showing expression of CHCHD2 in a minority of microglia. Scale bar: 10 μm. **d** Quantification of the percentage of CHCHD2 positive cells by cell type in Iba1, GFAP or NeuN positive cells. **e** and **f** Immunofluorescence staining images of *substantia nigra* of control and PD brain slices probed with the antibody against CHCHD2 (green) and TH (red). Scale bar = 20 μm. **g** Quantification of TH^+^ and CHCHD2^+^ cells in *substantia nigra*, demonstrating lower numbers of surviving neuronal cells and surviving CHCHD2^+^ cells in PD patients. *p* < 0.001, multiple t-test, n = 3 each. **h** Quantification of immunofluorescent signal demonstrating reduced CHCHD2 immunofluorescent intensity in *substantia nigra* (*p* < 0.001, Mann–Whitney U test, n = 3)

To probe whether expression of CHCHD2 is also reduced in the central nervous system (CNS) of PD patients, its expression was examined in the post-mortem *substantia nigra* slices obtained at autopsy from the brains of PD patients, along with age-matched controls, as well as in the animal model. Firstly, we examined the expression pattern of CHCHD2, both in the *substantia nigra* of human and wild type C57BL mice. Co-labeling of CHCHD2 with a pan-neuronal marker (NeuN) and a dopaminergic neuronal marker (Tyrosine hydroxylase, TH) revealed that CHCHD2 is expressed in nearly all neurons (> 90%, Fig. 2a for human, Fig. S2A for mice). CHCHD2 is also expressed in > 90% astrocytes (Fig. 2b for human, Fig. S2B for mice), but only in 10–30% microglia cells (Fig. 2c for human, Fig. S2C for mice). Subcellularly, CHCHD2 is located in mitochondria, as indicated by co-localization with Translocase of Outer Mitochondrial Membrane 20 (TOMM20) (Fig. S2D). Consistent with previous reports [19, 27], loss of dopaminergic neurons, indicated by TH staining, was detected (Fig. 2e–g, 13.2 ± 1.3 in Ctl vs. 4.6 ± 0.6 in PD per field, *p* < 0.001, multiple t-test). The number of CHCHD2 positive cells was also significantly lower, with 5.46 ± 0.6 per field in PD patients versus 15.2 ± 1.6 neurons in control (Fig. 2e–g, *p* < 0.001, multiple t-test). Fluorescence intensity of CHCHD2 protein in the surviving neurons of *substantia nigra* of PD patients also decreased markedly (Fig. 2e–f and h *p* < 0.001, Mann–Whitney U test).

**Fig. 3:**
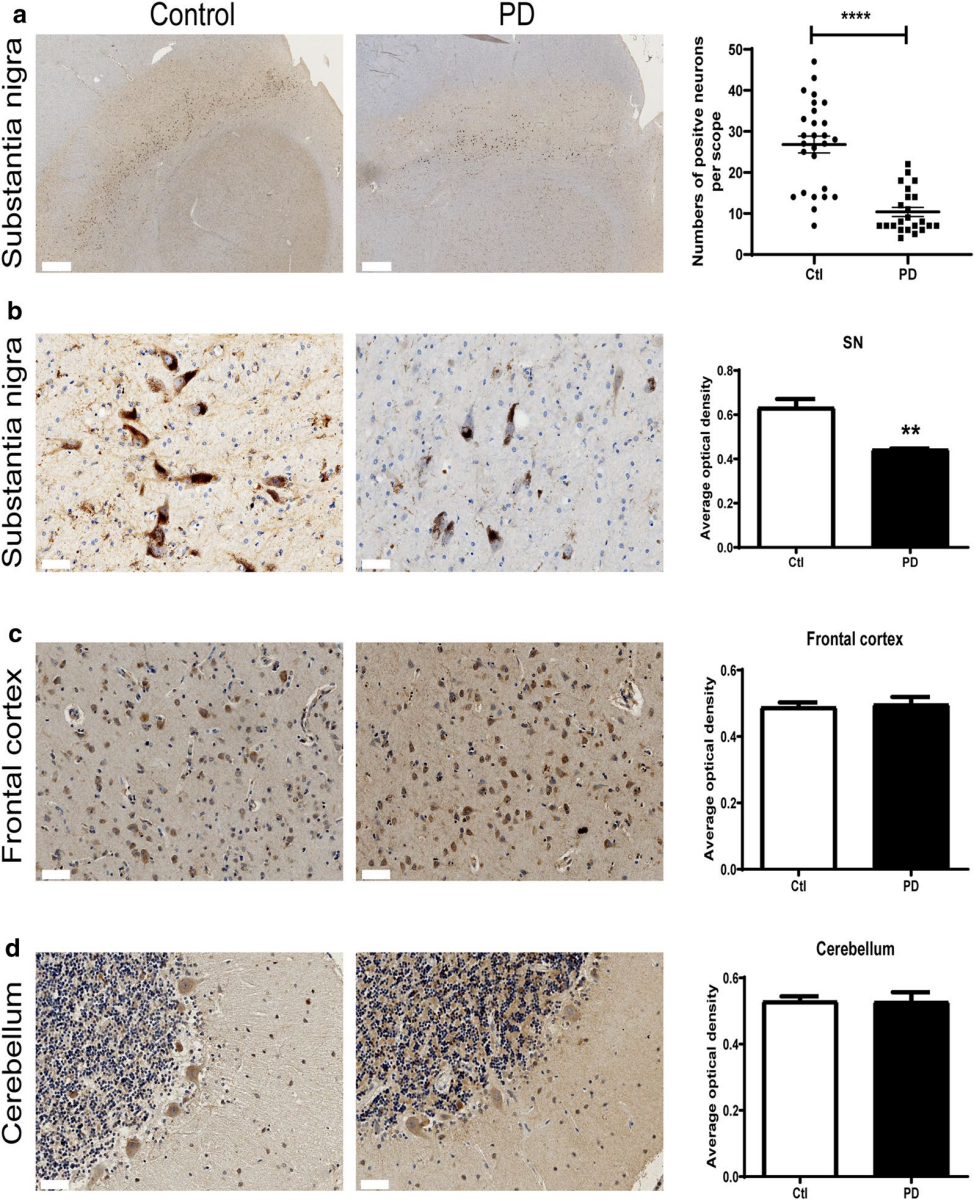
CHCHD2 expression was reduced specifically in the *substantia nigra* of PD patients when examined by immunohistochemical staining. **a** Reduced number of CHCHD2 positive cells in *substantia nigra* of PD patients. Whole slice scanned images of control and PD patients were shown in left and middle panel. Scale bar: 1000 μm. Statistics data of the number of CHCHD2 positive cells was shown in right panel. *p* < 0.0001, Mann–Whitney U test. **b** The immunohistochemical staining intensity of CHCHD2 was reduced in *substantia nigra* of PD patients. *p* < 0.01, Mann–Whitney U test, n = 3. Scale bar: 50 μm. **c** CHCHD2 expression was not altered in frontal cortex. *p* > 0.05, Mann–Whitney U test, n = 3. Scale bar: 50 μm. **d** CHCHD2 expression was not altered in cerebellum. *p* > 0.05, Mann–Whitney U test, n = 3. Scale bar: 50 μm

We also detected the expression of CHCHD2 in post-mortem slices across different brain regions by immunohistochemistry. Consistent with the results obtained by immunofluorescence in Fig. 2e–h, the number of CHCHD2 positive cells (Fig. 3a, *p* < 0.0001, Mann–Whitney U test) and density of CHCHD2 in the surviving cells (Fig. 3b, *p* < 0.01, Mann–Whitney U test) of *substantia nigra* of PD patients were reduced significantly, compared with control. In contrast, the expression of CHCHD2 in frontal cortex (Fig. 3c, *p* > 0.05, Mann–Whitney U test) and cerebellum (Fig. 3d, *p* > 0.05, Mann–Whitney U test) were not changed. These results suggested CHCHD2 was preferentially reduced in the *substantia nigra* of PD patients.

### Reduced mRNA and protein expression of CHCHD2 in erythrocytes and brains of A53T α-synuclein mice

**Fig. 4:**
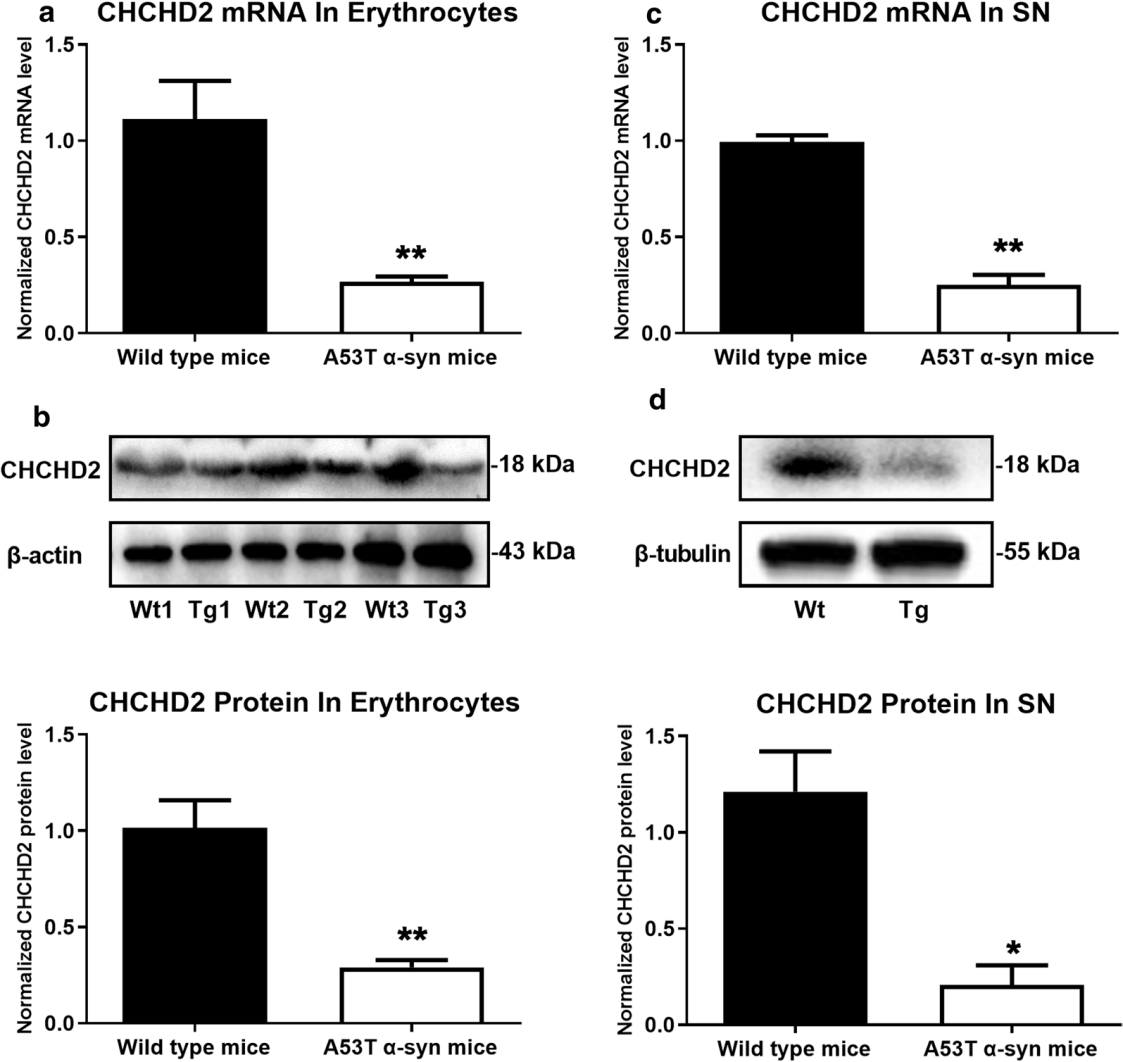
Reduced expression of CHCHD2 in the erythrocytes and *substantia nigra* of A53T α-synuclein transgenic mice. **a** Compared to non-transgenic wild type mice, normalized mRNA of CHCHD2 in erythrocytes of A53T α-synuclein transgenic mice was significantly decreased from 1.11 ± 0.20 to 0.27 ± 0.03 (*p* < 0.01, Mann–Whitney U test, n = 6). **b** Western blot results showed a significantly reduced protein expression of CHCHD2 in erythrocytes of transgenic mice (*p* < 0.01, Mann–Whitney U test, n = 3). Upper: representative Western blot images. β-actin was used as an internal control. Wt: non-transgenic wild type mice. Tg: A53T transgenic mice. Lower: quantification of Western blots. **c** Real-time qPCR also detected a reduced mRNA expression of CHCHD2 in *substantia nigra* of transgenic mice (*p* < 0.01, Mann–Whitney U test, n = 6). **d** CHCHD2 protein was decreased from 1.21 ± 0.21 in *substantia nigra* of wild type mice to 0.21 ± 0.10 in *substantia nigra* of transgenic mice (*p* < 0.05, Mann–Whitney U test, n = 3). Upper: representative Western blot images. β-tubulin was used as an internal control. Lower: quantification of Western blots. Of note, the sample number (N) is different because mRNA and proteins were measured in two different sets of animals

Having observed both CNS and peripheral reduction of CHCHD2 expression in PD patients, we next sought to explore the potential underlying mechanisms in a PD mouse model. For this purpose, we used the A53T α-synuclein transgenic mouse model (M83 line), an extensively utilized model where progressive pathology and behavior are driven by expression of mutated α-synuclein [8]. A53T^+/+^ mice were used at 10 months old, when neurologic defects are readily detectable. As shown in Fig. 4, the relative mRNA and protein expression of CHCHD2 in erythrocytes was significantly lower in A53T^+/+^ mice (0.27 ± 0.03 for mRNA, *p* < 0.01, Mann–Whitney U test, n = 6, Fig. 4a; and 0.29 ± 0.04 for protein, *p* < 0.01, Mann–Whitney U test, n = 3, Fig. 4b) compared to wild type control mice (1.11 ± 0.20 for mRNA; 1.02 ± 0.14 for protein). In the *substantia nigra*, real time PCR and Western blot results clearly showed that the mRNA (Fig. 4c, *p* < 0.01, Mann–Whitney U test, n = 6) and protein expression (Fig. 4d, *p* < 0.05, Mann–Whitney U test, n = 3) of CHCHD2 were significantly decreased.

**Fig. 5:**
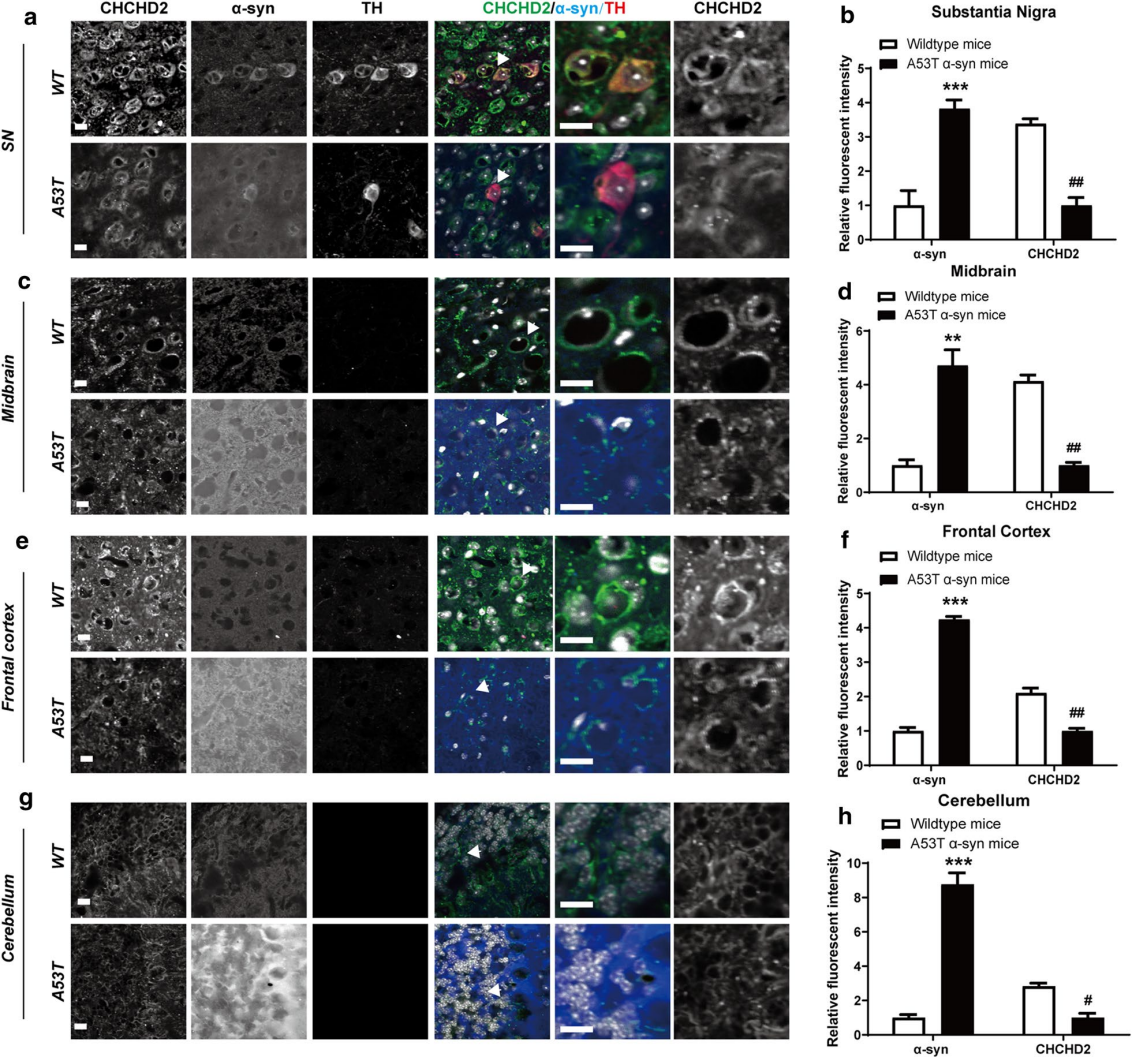
Immunofluorescent staining of CHCHD2 and α-synuclein in different areas of mouse brain slices. **a** and **b** Decreased CHCHD2 (green) and increased α-synuclein (blue) in *substantia nigra* (***p < 0.001, ^##^*p* < 0.01, Mann–Whitney U test, n = 3). **c** and **d** Decreased CHCHD2 (green) and increased α-synuclein (blue) in rest of midbrain (**p < 0.01, ^##^*p* < 0.01, Mann–Whitney U test, n = 3). **e** and **f** Decreased CHCHD2 (green) and increased α-synuclein (blue) in frontal cortex (***p < 0.001, ^##^*p* < 0.01, Mann–Whitney U test, n = 3). **g** and **h** Decreased CHCHD2 (green) and increased α-synuclein (blue) in cerebellum (***p < 0.001, ^#^*p* < 0.05, Mann–Whitney U test, n = 3). Scale bar: 10 μm

We also detected a reduced expression of CHCHD2 by immunofluorescence across several brain regions, including *substantia nigra* (Fig. 5a, b), the rest of midbrain (Fig. 5c, d), frontal cortex (Fig. 5e, f) and cerebellum (Fig. 5g, h) in A53T^+/+^ mice compared to non-transgenic wild type mice (all *p* <0.05, Mann–Whitney U test, n = 3). Consistent with previous reports [8, 10, 35], aggregated α-synuclein was increased in these regions (all *p* < 0.05, Mann–Whitney U test, n = 3). A correlation analysis indicated a negative correlation between the expression of CHCHD2 and the level of aggregated α-synuclein (r = − 0.978, *p* < 0.001 in *substantia nigra*, Fig. S3A; r = − 0.943, *p* < 0.01 in the rest of midbrain, Fig. S3B; r = − 0.952, *p* < 0.01 in frontal cortex, Fig. S3C; r = − 0.978, *p* < 0.001 in cerebellum, Fig. S3D).

### Reduced mRNA and protein expression of CHCHD2 in a cellular model of PD

**Fig. 6:**
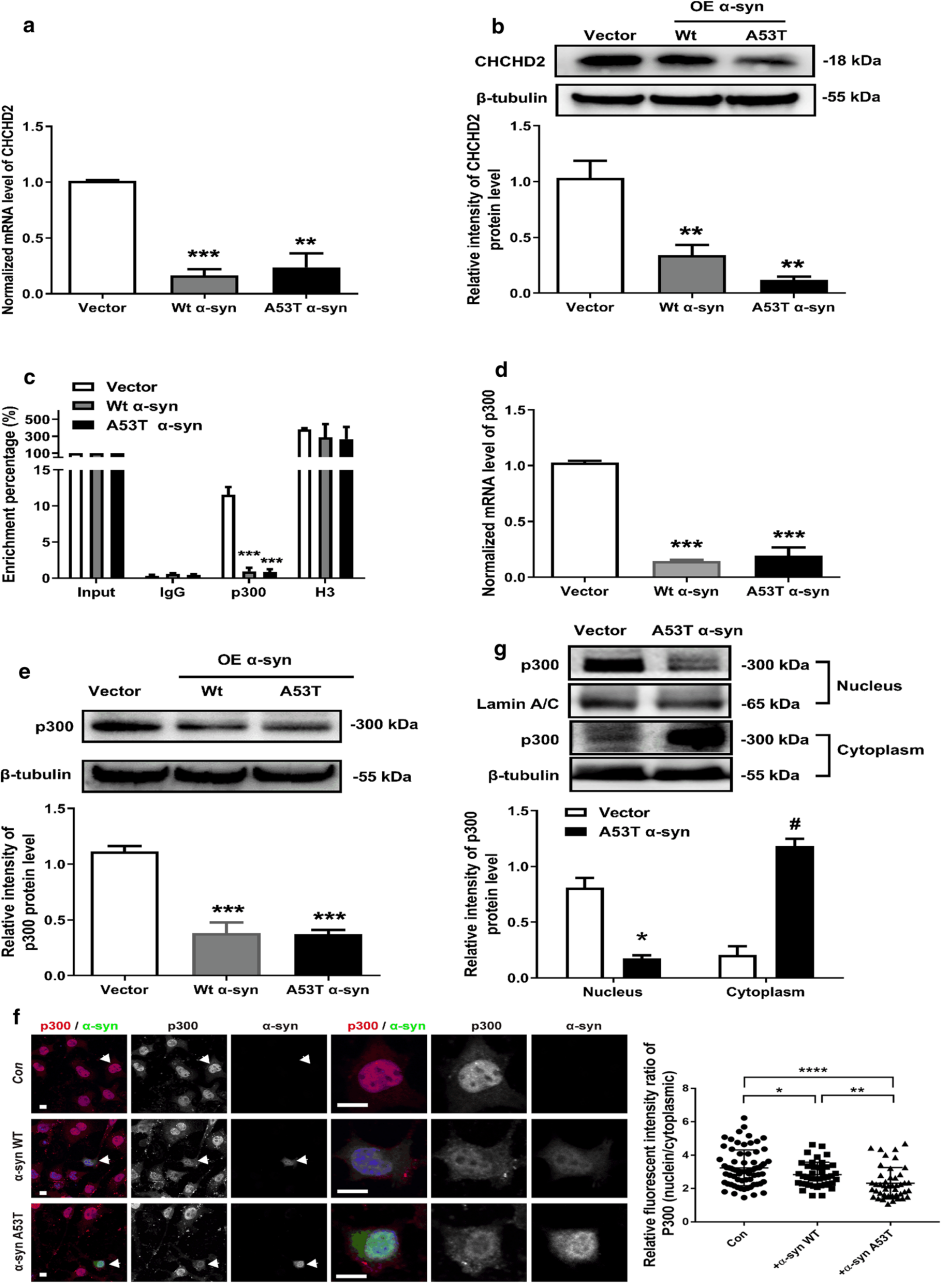
Overexpression of α-synuclein reduced CHCHD2 expression in MN9D cells, possibly by altering the expression and subcellular localization of p300. **a** Reduced mRNA expression of CHCHD2 in MN9D cells after overexpression of α-synuclein (F(2, 6) = 35.31, one-way ANOVA, n = 3; *p* < 0.001 for Wt α-syn vs. Vector, *p* < 0.01 for A53T α-syn vs. Vector). **b** Reduced protein expression of CHCHD2 in MN9D cells after overexpression of α-synuclein [F(2, 6) = 20.75, one-way ANOVA, n = 3; *p* < 0.05 for Wt or A53T α-syn vs. Vector]. **c** Direct interaction between p300 and CHCHD2 promoter as revealed by ChIP result. Overexpression of both wild type and A53T α-synuclein reduced the interaction of p300 and CHCHD2 promoter [F(2, 6) = 73.44,one-way ANOVA, n = 3; both *p* < 0.001, compared to Vector]. Histone 3 (H3) was used as positive control. **d** Decreased mRNA expression of p300 after overexpression of α-synuclein [F(2, 6) = 130.2, one-way ANOVA, n = 3; both *p* < 0.001, compared to Vector]. **e** Protein expression of p300 [F(2, 6) = 41.95, one-way ANOVA, n = 3; both *p* < 0.001, compared to Vector]. **f** Reduced nuclear distribution of p300 after overexpression of α-synuclein revealed by immunofluorescence (F (2, 154) = 12.8, one-way ANOVA, *p* < 0.05 or 0.01 or 0.0001). **g** Reduced localization of p300 in nucleus revealed by western blot (*p* < 0.05, Mann–Whitney U test, n = 3); Increased localization of p300 in cytoplasm (*p* < 0.05, Mann–Whitney U test, n = 3)

To further explore the potential mechanisms leading to decreased CHCHD2 expression in PD patients, wild type and A53T α-synuclein plasmids were transfected in MN9D cells, a line that is derived from dopaminergic cells and previously utilized in multiple in vitro PD models [5, 15, 37]. As expected, transfection of the cells with α-synuclein vector resulted in increased mRNA [Fig. S4A, *p* < 0.01, F(2, 6) = 20.95, one-way ANOVA, n = 3] and protein [Fig. S4B, F(2, 9) = 14.37, one-way ANOVA, n = 4; *p* < 0.01 for A53T vs. Vector] expression of α-synuclein. The overexpression of α-synuclein also dramatically reduced the number of CHCHD2 mRNA transcripts, regardless of whether the vector was wild type or A53T α-synuclein [Fig. 6a F(2, 6) = 35.31, one-way ANOVA, n = 3; *p* < 0.001 for Wt α-syn vs. Vector, *p* < 0.01 for A53T α-syn vs. Vector]. Consistent with the human data, the protein expression of CHCHD2 was also reduced markedly[Fig. 6b, F(2, 6) = 20.75, one-way ANOVA, n = 3; *p* < 0.05 for Wt or A53T α-syn vs. Vector].

### α-synuclein negatively regulates the expression of CHCHD2, possibly via modulation of P300

The results shown above suggest that reduced CHCHD2 protein is largely attributable to a decreased level of mRNA, likely secondary to overexpression of α-synuclein. To further investigate potential links between α-synuclein overexpression and CHCHD2 level, we asked whether there is a direct interaction of α-synuclein with the promoter of CHCHD2. Initial chromatin immunoprecipitation (ChIP) experiments failed to demonstrate any direct interaction between α-synuclein and CHCHD2 promoter (Additional file 1: Fig. S5A). Therefore, we next investigated whether α-synuclein could negatively regulate the expression of CHCHD2 indirectly. Because it has previously been shown that α-synuclein could negatively regulate protein kinase C expression by reducing the expression and activity of p300 histone acetyltransferase [13], we tested whether there is a direct interaction between p300 and the promoter of CHCHD2. As shown in Fig. 6c, ChIP results distinctly showed that p300 can directly bind the promoter of CHCHD2. Additionally, overexpression of wild type or mutant α-synuclein in MN9D cells reduced the interaction of p300 with the promoter of CHCHD2 (*p* < 0.001, F (2, 6) = 73.44, one-way ANOVA, n = 3).

We next examined the potential mechanisms by which α-synuclein expression alters the function of p300, by measuring the effect of α-synuclein overexpression on p300. Significant reductions in the mRNA (Fig. 6d, *p* <0.001, F(2, 6) = 130.2, one-way ANOVA, n = 3) and protein expression (Fig. 6e, *p* <0.001, F(2, 6) = 41.95, one-way ANOVA, n = 3) of p300 were seen 48h after transfection of α-synuclein vector. Direct protein interaction between p300 and α-synuclein was also demonstrated by reciprocal co-immunoprecipitation experiments (Additional file 1: Fig. S5B). Immunofluorescence results showed that p300 was mainly expressed in the nucleus, while it was less expressed in the cytoplasm of vector transfected Mn9D cells. However, overexpression of wild type or A53T α-synuclein significantly reduced the nuclear expression of p300 (Fig. 6f, *p* < 0.0001, F(2, 154) = 12.8, one-way ANOVA). Western blot of the protein of isolated subcellular fractions showed an altered distribution, with a higher proportion of p300 in the cytoplasm, compared to a lower amount in the nucleus after α-synuclein (A53T) overexpression compared to vector-transfected cells (Fig. 6g, *p* < 0.05, Mann–Whitney U test, n = 3). All the above results suggested that α-synuclein may inhibit the expression of CHCHD2, possibly by reducing the expression and the nuclear distribution of p300.

### Exploration of erythrocytic CHCHD2 as a PD biomarker in a large cohort

**Fig. 7:**
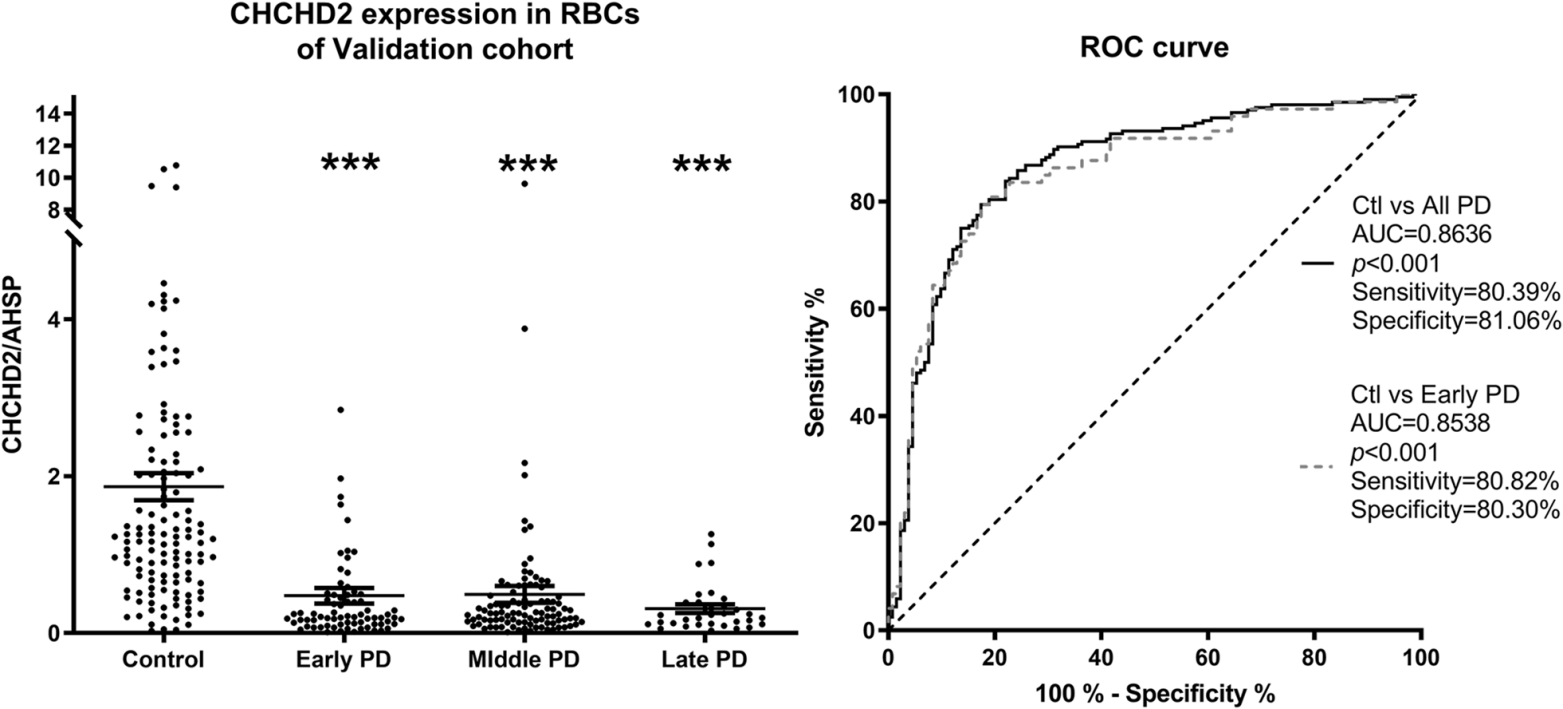
Reduced CHCHD2 mRNA in erythrocytes predicts PD. **a** Reduced CHCHD2 mRNA in erythrocytes of PD patients was further confirmed in a larger, validation cohort of 135 normal controls, 73 Early PD, 98 Mid PD and 34 Late PD patients [*p* < 0.001 each, F (3, 332) = 26.11, one-way ANOVA]. No difference in CHCHD2 mRNA expression was determined among PD groups by Tukey’s multiple comparisons test. **b** ROC analysis revealed diagnostic values of 80.4% sensitivity and 81.1% specificity of CHCHD2 for PD diagnosis

Having demonstrated that expression of CHCHD2 is significantly reduced in the *substantia nigra* as well as in erythrocytes in PD patients, we turned our attention back to its utility as a more convenient biomarker for PD diagnosis. To achieve this goal, we further examined CHCHD2 mRNA in erythrocytes of individual PD patients in a cohort of 340 subjects including patients with early- (n = 73), middle- (n = 98), and late-stage PD (n = 34) along with controls (n = 135). As shown in Fig. 7a, CHCHD2 mRNA was reduced significantly in all PD groups compared to controls, as detected by ddPCR [F (3, 332) = 26.11, one-way ANOVA; *p* < 0.001 for each PD group vs. control], consistent with our discovery cohort results.

Because PD is an age-related disorder, not evenly distributed by sex, we next investigated the impact of both sex and age dependence on CHCHD2 mRNA levels isolated from erythrocytes. Two-way ANOVA comparisons of male and female control and PD patient samples revealed no significant difference in CHCHD2 mRNA levels between sexes [Additional file 1: Fig. S6A, *p* = 0.65, F (1, 326) = 0.2077], while linear regression analysis revealed no correlation between CHCHD2 mRNA levels and age in either group (Additional file 1: Fig. S6B, control: *p* = 0.49, R = 0.06; PD: *p* = 0.35, R = − 0.07).

We also evaluated CHCHD2 mRNA as a diagnostic marker in erythrocytes. Analysis of Receiver Operating Characteristic (ROC) curve was performed to evaluate the diagnostic accuracy of CHCHD2 mRNA levels in the total PD cohort as well as PD patients at early-stage. The results were similar for both analyses, and when the specificity was anchored to ≥ 80%, sensitivity for detecting early PD versus healthy controls was 80.82%, yielding a final diagnostic value of 85.38% for PD vs. controls (Additional file 1: Table S2 and Fig. 7b).

Finally, we compared the CHCHD2 mRNA levels in erythrocytes with disease severity, including worsening of motor symptoms and mild cognitive impairment (MCI), and disease duration by comparing CHCHD2 mRNA to the UPDRS part III on-state motor scores, Montreal Cognitive Assessment (MoCA) score, and time following diagnosis in the validation cohorts of PD patients (n = 205 total; cases < 50 years were not excluded due to the lack of age dependence as determined above). Once again, as determined by linear regression analyses, no significant association could be determined between CHCHD2 mRNA levels and the disease severity (*p* = 0.49, R = − 0.05 for UPDRS motor), disease duration (*p* = 0.62, R = 0.04) or MOCA scores (*p* = 0.11, R = 0.11) (Additional file 1: Fig. S6C–E).

## Discussion

The CHCHD2 gene is a recently discovered PD causative gene that drives development of PD with autosomal dominant inheritance [6]. More recently, mutations in CHCHD2 have also been found in sporadic PD [29, 39, 46]. CHCHD2 protein is localized to mitochondria, which are intimately associated with PD pathogenesis [48]. It has been demonstrated that the loss of CHCHD2 causes abnormal matrix structures and impaired oxygen respiration in mitochondria, leading to oxidative stress, dopaminergic neuron loss and motor dysfunction in *Drosophila* [22]. Additionally, knockout of CHCHD2 or CHCHD10 in human induced pluripotent stem cells led to increased proton leakage and respiration, as well as loss of synaptic function [11]. CHCHD2/CHCHD10 double knockout mice showed disrupted mitochondrial cristae [17].

Two major observations of the current study are: (1) the expression of CHCHD2 was reduced significantly in human erythrocytes, *substantia nigra* of brain collected at autopsy, and mice carrying transgenic mutant A53T α-synuclein; and (2) the expression of CHCHD2 in erythrocytes, a readily accessible clinical sample source, could be an early diagnostic marker of PD.

Using NanoString multiplex gene expression analysis, among a total of 21 genes screened, CHCHD2 mRNA was discovered to be consistently reduced in various stages of PD patients. The corresponding protein was also reduced in erythrocytes (Fig. 1). The mechanisms responsible for the CHCHD2 reduction in the erythrocytes of PD patients are unknown, especially given that mature erythrocytes are incapable of transcriptional regulation. One possibility is that the transcriptional regulation occurred in bone marrow or at the stage of reticulocytes, a data point not collected in the current study. However, reticulocytes account for 0.5–2.5% of red blood cells [38], and there is no evidence to suggest that there is a difference between PD and controls in reticulocyte counts. Therefore, future study needs to include investigations in bone marrow or at least to consider the ratio of erythrocytes/reticulocytes in various cohorts.

One surprising aspect of this study is that while reduced CHCHD2 expression was observed in (and correlated between) both the *substantia nigra* (Figs. 2 and 3) and periphery of human PD patients, the reduction was not universal within the CNS, as it did not extend to the frontal cortex or cerebellum. In contrast, reduced CHCHD2 mRNA and protein expression were detected in the *substantia nigra*, prefrontal cortex and cerebellum of A53T α-synuclein transgenic mice (Figs. 4 and 5). The discrepancy in affected brain region between human and mice studies may have been due to the different expression patterns of pathological α-synuclein species in human and mice; specifically, the PD patients used in our study were at Braak Stage 3, a stage at which Lewy body pathology is present in the *substantia nigra,* but not the cortex or cerebellum. In contrast, transgenic α-synuclein is widely expressed in the central nervous system in the mouse model (M83 line), without the progressive spread observed in human patients. Whether lower expression of CHCHD2 in other brain regions becomes more widespread in the human brain as the disease progresses remains to be determined. Moreover, as with other gene mutations associated with PD, e.g., *SNCA* and *LRRK2*, though mutations occur systematically, dopaminergic neurons are preferentially vulnerable. Why and how CHCHD2 mutations/reductions result specifically in dopaminergic neurodegeneration needs to be investigated further.

Within affected brain regions, compared to healthy controls, both the number of CHCHD2 positive neurons and its level of expression in surviving neurons were reduced in the *substantia nigra* of PD patients (Figs. 2 and 3), suggesting the possibility of an interaction between PD pathological processes (e.g., accumulation of pathological α-synuclein species) and CHCHD2 reduction. Consistent with this hypothesis, correlation analysis revealed a negative correlation between protein expression levels of α-synuclein and CHCHD2 in mice (Additional file 1: Fig. S3), i.e., reduced CHCHD2 level may be driven by increased levels of pathogenic α-synuclein.

How might α-synuclein, especially pathogenic forms, inhibit the expression of CHCHD2? A previous study indicated that α-synuclein negatively regulates protein kinase C expression by reducing the expression and activity of p300 histone acetyltransferase [13]. Consistent with this observation, overexpression of wild type and A53T mutant α-synuclein in MN9D cells, a mouse dopaminergic cell line, significantly reduced the mRNA and protein expression of CHCHD2, with the effect being more significant when A53T mutant α-synuclein was used (Fig. 6). Additionally, direct interaction of p300, instead of α-synuclein, with the promoter of CHCHD2 gene was detected by ChIP experiment whereas overexpression of wild type or A53T mutant α-synuclein reduced their interaction. However, α-synuclein and CHCHD2 may have a more complicated mutual interaction. For example, in a recent study, mutations in CHCHD2 resulted in α-synuclein aggregation [12]. Further, although a link between the reduced expression of CHCHD2 and nuclear distribution of p300 is clearly implicated (Fig. 6), the direct involvement of p300 needs to be confirmed by additional studies, e.g., knockdown of its expression in a cellular model of PD.

Regardless of the mechanism(s) underlying CHCHD2 expression in PD patients, reduced CHCHD2 mRNA in all stages of PD was validated by ddPCR in a larger cohort, suggesting that levels of CHCHD2 mRNA may act as a biomarker for detecting PD using patient blood, even at early disease stage, with a sensitivity and specificity of 80 and 81%, respectively (Fig. 7). Indeed, the level of CHCHD2 was not correlated with disease severity, motor or cognition, suggesting a possible flooring effect, possibly because the changes occur early during the disease process. In addition, no significant correlation was observed between CHCHD2 mRNA levels and sex or age (Additional file 1: Fig. S6). The result is quite significant in that reduced CHCHD2 mRNA could be a potential biomarker for early PD, when clinical diagnosis is most difficult due to overlapping clinical phenotypes in related diseases. However, to truly test this biomarker, future validation studies should include a reasonably large cohort of patients with multiple system atrophy (MSA) and/ or progressive supranuclear palsy (PSP). Additionally, moving forward, the biomarker should be tested in the premotor stage when nigrostriatal degeneration can be confirmed by DAT or PET imaging.

In summary, reduction in protein and mRNA expression of CHCHD2 is widespread in PD patients, from CNS to peripheral erythrocytes. The underlying mechanisms, though they remain to be investigated, are likely related to synucleinopathy and involvement of p300. CHCHD2 mRNA in erythrocytes, which is easily accessible, may serve as a convenient yet robust biomarker of PD, particularly for diagnosis of PD at early stages.

## Materials and methods

### Participants

This study was approved by the Institutional Review Boards of all participating institutions. Subjects for both the discovery (48 in total; 12 healthy controls, 36 PD patients) and validation (340 in total; 135 healthy controls, and 205 PD patients) studies were recruited from Beijing Tiantan Hospital between 2016 and 2018. All participants provided informed consent and underwent a neurologist-conducted evaluation that consisted of a structured interview, neurological examination, laboratory tests, and neuropsychological assessments. All control subjects were community volunteers who had Mini Mental State Exam Scores > 24, paragraph recall scores > 6, no history of neurological disease, and no history or evidence of cognitive or functional decline. All PD patients met UKPD Society Brain Bank clinical diagnostic criteria for PD [9]. PD patient samples were further categorized based on UPDRS Part III on-state motor scores to approximate disease stage, according to the method we reported previously [16]. That is, patients with UPDRS scores < 15 were defined as having early-stage PD, those with scores ranging from 15 to 30 were classified as middle-stage PD, while those with scores > 30 were classified as late-stage PD patients. Demographic data for all subjects and UPDRS scores for all PD patients used in the study are listed in Additional file 1: Tables S1 and S2.

### Animals

Transgenic mice expressing A53T (M83 line) human α-synuclein protein under the control of the prion protein promoter [8] were purchased from The Jackson Laboratory. Non-transgenic mice with the same background were used as controls. All mice were housed in separate cages with free access to food and water. The room temperature was kept at 24 ± 1 °C under natural light–dark cycle. All animal experimental procedures were approved by the Animal Care and Use Committee of Peking University.

### RNA isolation from erythrocytes

To separate erythrocytes from plasma and buffy coat, fresh whole blood samples were immediately centrifuged after phlebotomy at 2000 g for 10 min. 200 μl of pure erythrocytes were then used to extract total RNA using Trizol reagent (Invitrogen, USA) according to the manufacturer’s protocol. RNA was assessed for quantity using Nanodrop 2000, and for quality using the 2100 Bioanalyzer (Agilent Technologies, Canada). RNA was either used immediately or stored at − 80 ℃ consistently within experiments.

### NanoString nCounter method

The nCounter Analysis System (NanoString Technologies, Seattle, WA) allows for multiplexed digital mRNA profiling without amplification or generation of cDNA [7]. A total of 21 PD related genes [APOE (NM_000041.2), APP (NM_000484.3), ATP13A2 (NM_001141974.1), CHCHD2 (NM_016139.2), EIF4G1 (NM_004953.3), FBXO7 (NM_001033024.1), GATA1 (NM_002049.2), GBA (NM_001005742.2), LRRK2 (NM_198578.3), MAPT (NM_016834.3), PARK2 (NM_004562.2), PARK7 (NM_001123377.1), PINK1 (NM_032409.2), PLA2G6 (NM_001199562.1), PSEN1 (NM_000021.2), PSEN2 (NM_000447.2), SNCA (NM_000345.2), SNCAIP (NM_001242935.2), TPPP (NM_007030.2), UCHL1 (NM_004181.3), VPS35 (NM_018206.4)] were screened. Total RNA (100 ng) was hybridized with the Tagset probes and loaded into the nCounter prep-station, and then quantified using the nCounter Digital Analyzer. The NanoString platform includes negative control probes (not complementary to any endogenous mRNA) to assess background noise associated with the fluorescent barcode optical recognition system. Raw probe counts were normalized to a panel of three endogenous control genes [AHSP (NM_016633.2), β-actin (NM_001101.2), and GAPDH (NM_002046.3)] by taking the ratios of each gene’s counts per sample to the average across all samples and scaling by the median of these ratios in each sample. This normalization factor was also applied to the negative control probes counts. A detection threshold was defined for each sample as five times the mean of the negative control probe normalized counts.

### Digital droplet PCR quantification

For absolute quantification of mRNA of CHCHD2 in erythrocytes, we used the recently developed digital droplet PCR [25]. TaqMan probes (250 nM final concentration) and related primers set for CHCHD2 (labeled with a FAM at the 5′ end, 900 nM primers at final concentration) and AHSP (labeled with a VIC at the 5′ end, 150 nM primers at final concentration) were purchased from Thermo Fisher Scientific, Inc. (USA). AHSP, which is expressed mainly in erythrocytes and did not change in PD based on our NanoString data, was chosen as an internal control. We used SuperScript™ III Platinum™ One-Step qRT-PCR Kit (#11732020, Thermo Scientific, USA) for cDNA synthesis and PCR amplification performed in a single tube following the instructions of the manufacturer. The RainDrop Source emulsion generator (RainDance Technologies, Inc.) was used to generate emulsified micro droplets. Each 25 μl reaction system consisted of the 24 μl TaqMan gene expression ddPCR system and 1 μl of 25 × droplet stabilizer. After emulsion, the tube was directly sealed and put into an ABI ProFlex™ thermalcycler (Thermo Fisher Scientific, Inc. USA). PCR amplification program was as follows: 50 ℃, 15 min; 95 ℃, 4 min; 45 cycles of (95 ℃, 15 s; 60 ℃, 45 s). The heating and cooling rates of the thermalcycler were adjusted to 0.6 ℃/s for better PCR amplification in millions of micro droplets. After amplification, the tube was transferred to Raindrop Sense machine and the numbers of amplified droplets with suitable targets were analyzed with RainDrop Analyst v3 software.

### Western blot

Proteins were extracted from erythrocytes, cell lines, or tissues using SDS lysis buffer (2% SDS, 10% glycerol, 0.1 mM dithiothreitol and 0.2 M Tris–HCl, pH 6.8). Protein samples were resolved by SDS–polyacrylamide gel electrophoresis, transferred to polyvinylidene difluoride membrane and blotted with respective primary antibodies at 4 ℃ over night. The blots were washed in TBST and then incubated in horseradish peroxidase–conjugated goat anti-rabbit/mouse IgG secondary antibody. Protein bands were visualized using an enhanced chemiluminescence detection kit followed by autoradiography using Hyperfilm MP.

### Real-time PCR

The levels of mRNA for CHCHD2, α-synuclein or p300 in mice or cell lines were measured by real-time PCR. Briefly, total RNA was isolated from brain tissues or cell lines using Trizol™ (Life technologies, USA). Synthesis of first-strand cDNA was performed by reverse transcription of 1.0 μg total RNA using RT-PCR kit according to the manufacturer’s protocol (Takara Inc, Japan). Oligonucleotide primers corresponding to cDNA for mouse CHCHD2/α-synuclein/p300/GAPDH can be seen in Additional file 1: Table S3. The specificity for each primer set was confirmed by both electrophoresis of the PCR products on a 2.0% agarose gel and analyzing the melting (dissociation) curve using a 7500 ABI PRISM Sequence Detector System according to the manufacturer's instructions (Applied Biosystems) after each real-time PCR reaction. The relative amount of transcripts was calculated using the 2^−ΔΔCT^ method [33] and normalized to the endogenous reference gene GAPDH.

### Immunofluorescence staining of human and mouse brain tissues

The post-mortem human brain material was obtained from China National Health and Disease Human Brain Tissue Resource Center (Hangzhou). All materials have been collected from donors who provided written informed consent for a brain autopsy and permitting the use of the material and clinical information for research purposes. Demographic data for all subjects used in the study are listed in Additional file 1: Table S4. The paraffin-fixed formaldehyde-embedded human or frozen mouse brain tissues were cut into 6–10 μm sections. The immunofluorescence staining of brain tissues was performed in a double-blinded manner. Brain slices were incubated overnight at 4 °C with primary antibodies diluted in blocking solution. Brain slices were then washed with washing buffer (0.1% Tween in PBS) and incubated with corresponding secondary antibodies diluted in PBS containing 0.3% of Triton X-100 for 3 h. After washing with PBS or washing buffer, brain slides were embedded in Vectashield medium or Vectashield medium with DAPI. Immunofluorescence images were captured at room temperature using a Zeiss Confocal Microscope under 20× or 40× magnification. Mouse monoclonal antibody to CHCHD2(66302–1-Ig, Proteintech, 1:50), rabbit monoclonal antibodies [EPR12763] to NeuN (ab177487, Abcam, 1:500), chicken polyclonal antibodies to GFAP (AB5541, Merck Millipore, 1:500), rabbit monoclonal antibodies [EPR16588] to Iba1(ab178846, Abcam, 1:500), rabbit monoclonal antibodies [EPR15581-54] to TOMM20 (ab186735, Abcam, 1:250), chicken polyclonal antibodies to Tyrosine Hydroxylase (ab76442, Abcam, 1:500) and rabbit monoclonal [MJFR-14-6-4-2] antibodies to α-synuclein aggregate (ab209538, Abcam, 1:200) were used in IF. Alexa Fluor 405, 488, 555 or 633 conjugated secondary antibodies used in IF were purchased from Thermo Fisher Scientific, Inc. IF signals were quantified using Image J software (NIH).

### Immunohistochemistry staining of human brain tissues

For histochemical analysis of post-mortem brain slices of normal subjects and PD patients, paraffin-embedded sections (6 µm) were stained with anti-CHCHD2 (66302-1-Ig, Proteintech, 1:500) or anti-TH (ab76442, Abcam, 1:500) antibodies. Quantitation of immunostaining was conducted using NIH image J software. The same image exposure times and threshold settings were used for sections from all groups. The immunohistochemical staining of brain tissues and quantification were performed in a double-blinded manner.

### Cell culture and transfection

The MN9D dopaminergic neuronal cell line (American Type Culture Collection, Manassas, Va., USA) was cultured in DMEM high glucose medium containing 10% fetal bovine serum (Gibco, Carlsbad, CA, USA) and 100 U/mL of penicillin (Invitrogen) and 100 μg/mL of streptomycin (Invitrogen). The cells were incubated at 37 °C in an incubator with a humidified atmosphere of 95% air and 5% CO_2_. MN9D cells were transfected with wild type or A53T human α-synuclein cDNA in the pGV219 vector. Since α-synuclein expression and oligomerization are dynamic in these clones, comparisons were made between clones of wild type and A53T that were maintained in parallel from DNA transfection.

### Immunofluorescence (IF) for cultured cells

Cultured cells were fixed with 4% paraformaldehyde for 10 min followed by permeabilization by 0.2% Triton X-100 for 3 min before staining. Subsequently, cells were incubated with a primary antibody at 4 °C overnight and a secondary antibody conjugated with Alexa Fluor (Thermo Fisher Scientific) was added for detection. Cell nuclei were counterstained with DAPI dye. Fluorescence microscopy was performed with a Zeiss Confocal Microscope. IF signals were quantitated using the Image J software (NIH).

### Chromatin immunoprecipitation

Control, wild type, or A53T human α-synuclein-overexpressing MN9D cells were collected for ChIP using a SimpleChIP® Enzymatic Chromatin IP Kit (Magnetic Beads) (#9003; Cell Signaling), according to the manufacturer’s instructions. In brief, MN9D cells were first fixed with 1% formaldehyde to cross-link protein and DNA. Cell lysates were then subjected to sonication for ChIP. ChIP was done using anti-p300 antibody (ab14984; Abcam) or control IgG (sc-2027; Santa Cruz Technology). Precipitated DNA fragments containing gene promoter of CHCHD2 were detected by qPCR. Primers can be seen in Additional file 1: Table S3.

### Statistical analysis

All analyses were performed with Prism 8.0 (GraphPad). Linear regression analysis was used to determine the relationships between age, PD severity, MOCA, disease duration and CHCHD2. One-way analysis of variance (ANOVA) followed by Tukey's post-hoc test or two-way ANOVA followed by Bonferroni post-hoc test was used for multiple comparisons. F values with their associated degrees of freedom (treatment, time, interaction and residual) were expressed as F_(df of treatment, time, interaction/residual)_ = F values (treatment, time, interaction) in two-way ANOVA, and F_(df of treatment, residual)_ = F values. Please note that a non-parametric test was used whenever the sample numbers were too small (N = 3–6) to test for normality. Nonparametric Mann–Whitney U test was used for the comparison of the mean values between two groups. The multiple t-test analysis was used to analyze "matched" or "paired" data from the Grouped format data table. Additionally, relationships between the analytes and age, sex, and UPDRS motor score were analyzed with bivariate correlation using Pearson’s correlation coefficients. Values with *p* < 0.05 were regarded as significant. Receiver operating characteristic (ROC) curves were used to calculate the relationship between sensitivity and specificity for PD disease group versus healthy control. The “optimum” cutoff value from the ROC curve is determined by anchoring the specificity (or sensitivity) to be ≥ 80%.

## Supplementary Information

**Additional file 1.**Supplementary data

## Data Availability

All data needed to evaluate the conclusions in the paper are present in the paper and the Additional file are available from authors upon request.
