# Supplementary material for: Reduced erythrocytic CHCHD2 mRNA is associated with brain pathology of Parkinson’s disease
Source: Acta Neuropathol Commun. 2021 Mar 8;9:37. doi: 10.1186/s40478-021-01133-6 (PMC7941904; doi:10.1186/s40478-021-01133-6)
Supplement: Supplementary file 1 — Additional file 1.Supplementary data [file 40478_2021_1133_MOESM1_ESM.docx]

**Additional file**

Fig. S1. mRNA expression frequency in erythrocytes of healthy control

Fig. S2. Co-staining of CHCHD2 with markers of different cell types in mouse brain

Fig. S3. Correlation analysis of protein expression of α-synuclein and CHCHD2 in α-synuclein wild type and mutant mice.

Fig. S4. Increased mRNA and protein expression of α-synuclein in MN9D cells after transfection.

Fig. S5. α-synuclein interacts with p300, instead of the promoter of CHCHD2.

Fig. S6. No correlation of CHCHD2 mRNA in erythrocytes with sex, age, or disease severity traits.

Table S1. Erythrocytes group data of patients and controls for discovery cohort

Table S2. Erythrocytes group data of patients and controls for validation cohort

Table S3. Primer sequences.

Table S4. Demographics of post-mortem control and PD patients.


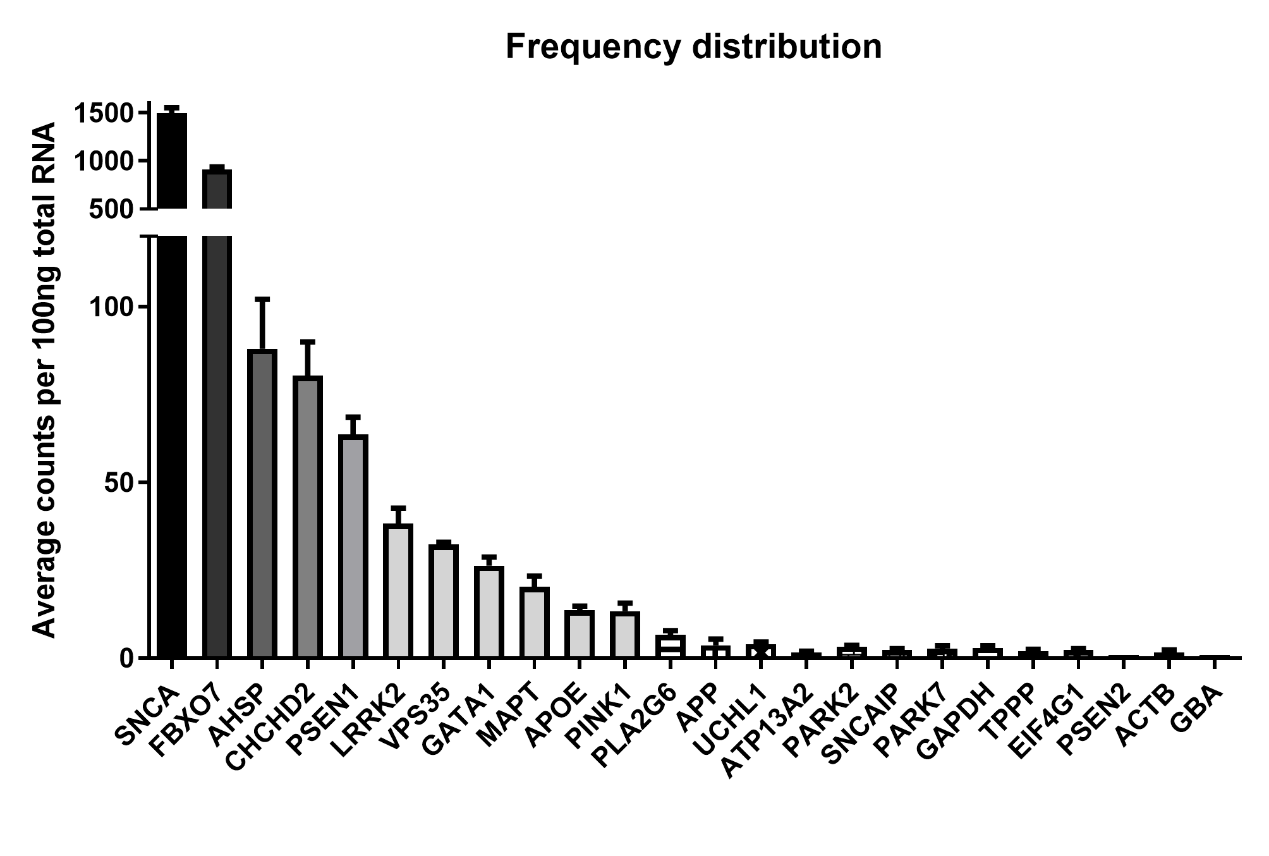


**Figure S1**. mRNA expression frequency in erythrocytes of healthy control.

Histogram of normalized mRNA counts of genes associated with PD or atypical Parkinsonism. Total RNA was extracted from erythrocytes of 3 pooled healthy control samples, and 100ng RNA was used for NanoString analysis.


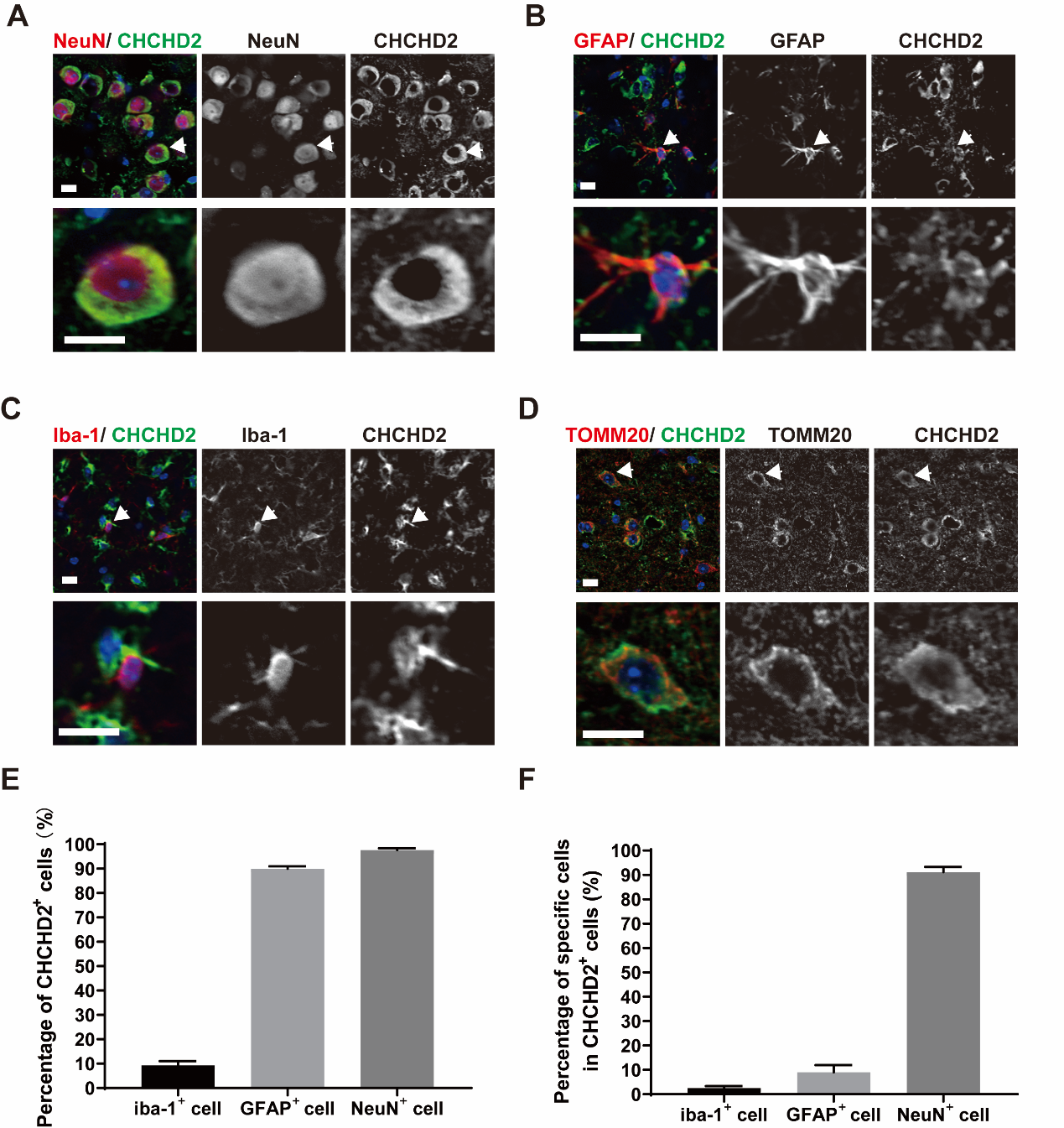


**Figure S2**. Localization of CHCHD2 in the brain of wild type mice.

**(A)**: Representative images of co-staining of CHCHD2 (green) with pan-neuronal marker NeuN (red). Scale bar: 10 μm.

**(B)**: Representative images of co-staining of CHCHD2 (green) with astrocyte marker GFAP (red). Scale bar: 10 μm.

**(C)**: Representative images of co-staining of CHCHD2 (green) with microglial marker Iba1 (red). Scale bar: 10 μm.

**(D)**: Co-staining of CHCHD2 (green) with TOMM20 (red). Scale bar: 10 μm.

**(E)**: Percentage of CHCHD2 positive cells in Iba1, GFAP or NeuN positive cells.

**(F)**: Percentage of Iba1, GFAP or NeuN positive cells in CHCHD2 positive cells.

**
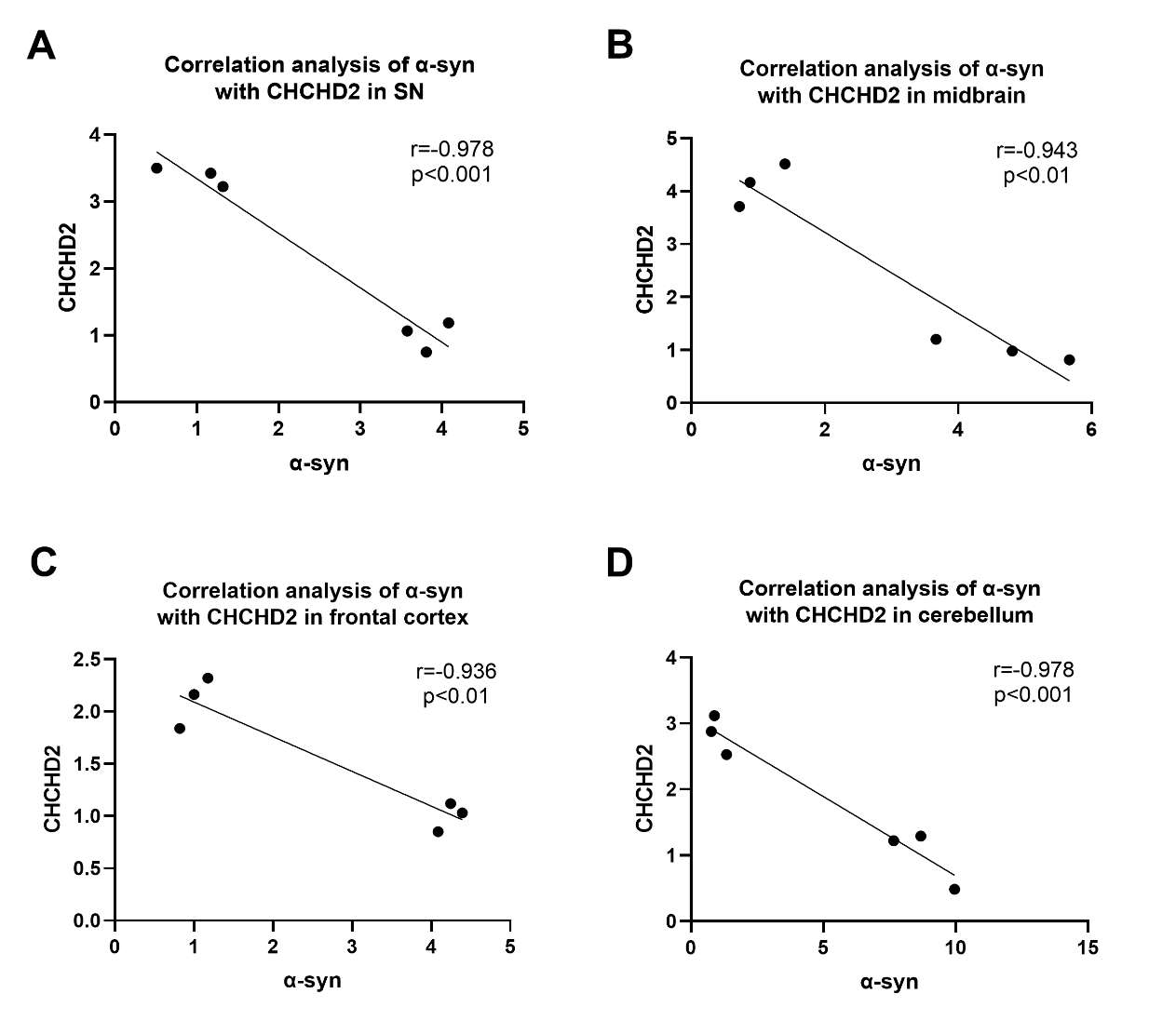
**

**Figure S3**. Correlation analysis of protein expression of α-synuclein and CHCHD2 in α-synuclein wild type and mutant mice. A: Correlation analysis revealed r=-0.978, p <0.001. B: Correlation analysis revealed r=-0.943, p <0.01. C: Correlation analysis revealed r=-0.952, p <0.01. D: Correlation analysis revealed r=-0.978, p <0.001.


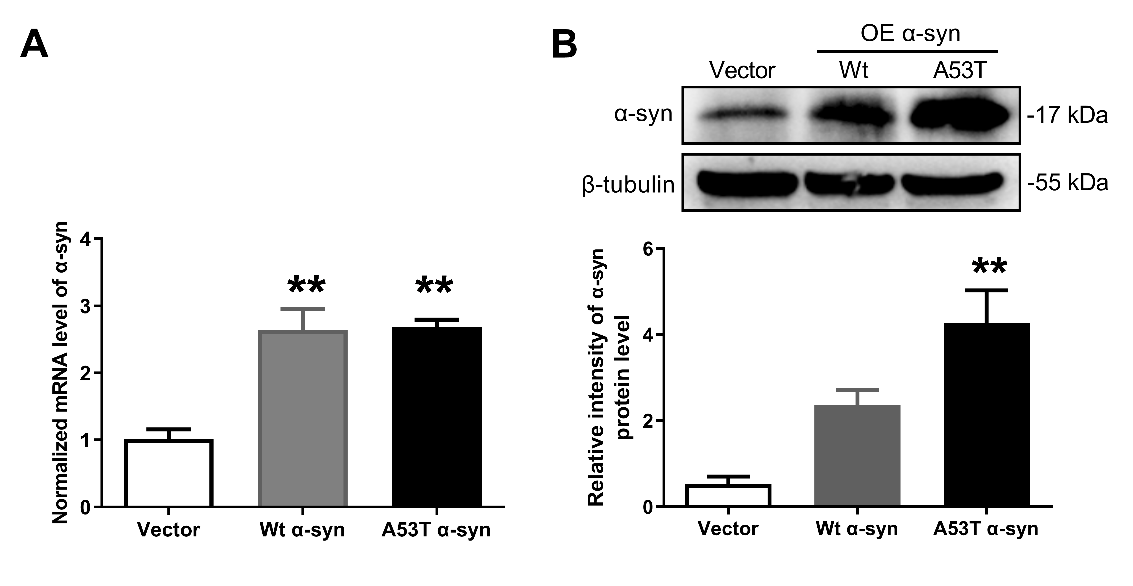


**Figure S4**. Increased mRNA and protein expression of α-synuclein in MN9D cells after transfection. A: Increased mRNA expression of wild type and A53T α-synuclein in MN9D cells (p <0.01, F(2, 6)=20.95, one-way ANOVA followed by Tukey's multiple comparisons test, n=3). B: Increased protein expression of α-synuclein in MN9D cells (p <0.01, F(2, 9)=14.37, one-way ANOVA followed by Tukey's multiple comparisons test, n=4).


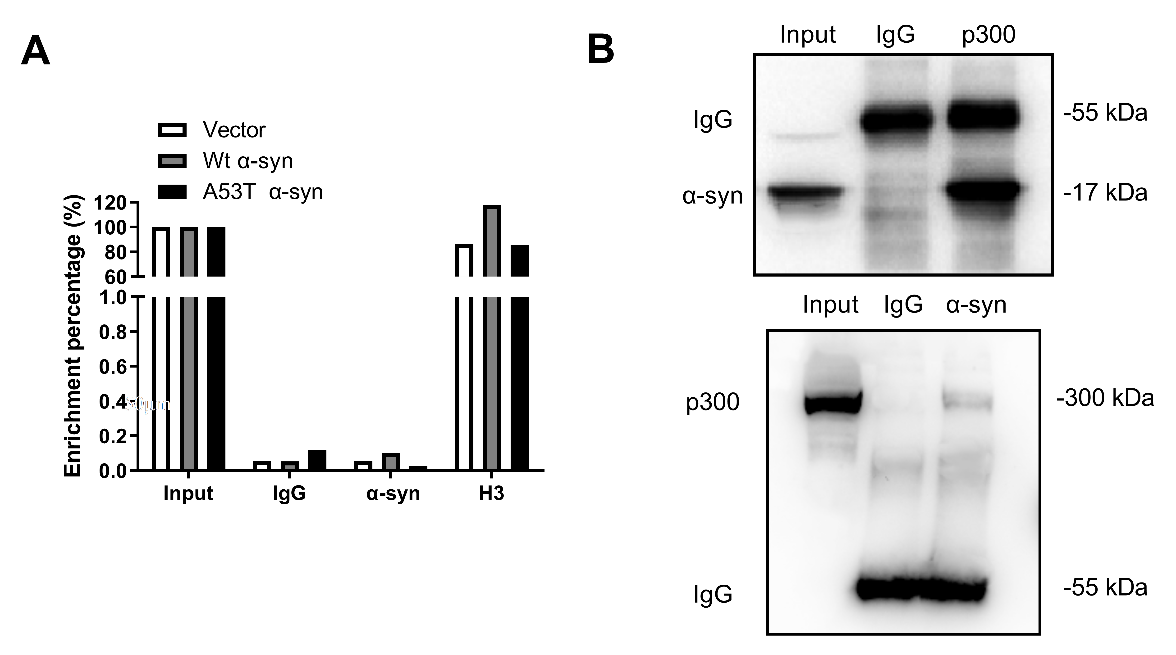


**Figure S5.** α-synuclein interacts with p300, instead of the promoter of CHCHD2. A: No direct interaction between α-synuclein and CHCHD2 promoter as revealed by ChIP. Histone 3 was used as positive control. B: Reciprocal co-immunoprecipitation experiments revealed interaction between α-synuclein and p300 (n=3). C: Co-localization of α-synuclein and p300 in Mn9D cells.


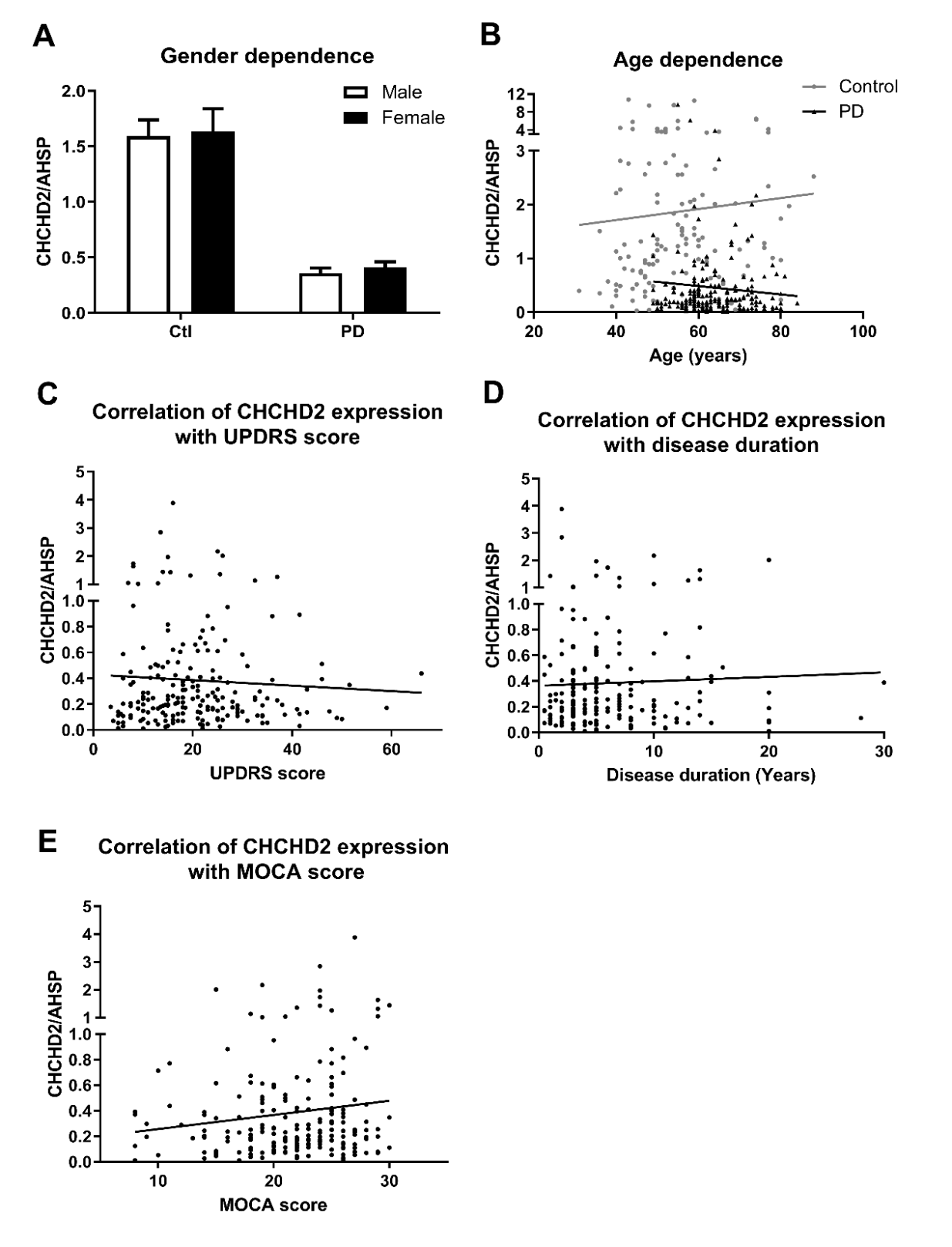


**Figure S6**. No correlation of CHCHD2 mRNA in erythrocytes with sex, age, or disease severity traits. A: CHCHD2 mRNA between male and female in controls or PD patients revealed no significant difference by two-way ANOVA (p = 0.65, F (1, 326) = 0.2077). B: No correlation between CHCHD2 mRNA and age dependence was detected by linear regression with Pearson’s coefficient across control (p = 0.49, r = 0.06) and PD (p = 0.35, r = -0.07). C-D: Plotting CHCHD2 mRNA levels against UPDRS motor scores, disease duration revealed no correlation between CHCHD2 mRNA and disease severity (UPDRS motor, p = 0.49 and r = -0.05), disease duration (p = 0.62 and r = 0.04). E: No correlation between CHCHD2 mRNA and MOCA scores (p = 0.11 and r = 0.11).

**Table S1.** Erythrocytes group data of patients and controls for discovery cohort

| **Sample** | **Group** | **Cases** | **Age**  (Mean±SD) | **Sex**  (M: F) | **UPDRS**  (Mean±SD) |
| --- | --- | --- | --- | --- | --- |
| **Control** | A | 4 | 65.2 ±10.8 | 2:2 | N/A |
|  | B | 4 | 66.2 ±12.0 | 2:2 |  |
|  | C | 4 | 67.7 ±8.3 | 2:2 |  |
| **Early PD** | A | 4 | 65.2 ±10.0 | 2:2 | 8.9 ±2.9 |
|  | B | 4 | 65.7 ±11.5 | 2:2 | 10.5 ±3.5 |
|  | C | 4 | 68.0 ±9.3 | 2:2 | 13.4 ±2.3 |
| **Mid PD** | A | 4 | 67.5 ±12.6 | 2:2 | 22.8 ±6.0 |
|  | B | 4 | 65.7 ±11.5 | 2:2 | 22.8 ±5.6 |
|  | C | 4 | 68.0 ±8.1 | 2:2 | 20.9 ±5.8 |
| **Late PD** | A | 4 | 66.0 ±10.3 | 2:2 | 40.4 ±7.2 |
|  | B | 4 | 65.7 ±11.2 | 2:2 | 39.3 ±6.2 |
|  | C | 4 | 68.2 ±6.9 | 2:2 | 39.8 ±4.6 |

**Table S2.** Erythrocytes group data of patients and controls for validation cohort

| **Sample** | **Cases** | **Age**  (Mean±SD) | **Sex**  (M:F) | **UPDRS**  (Mean±SD) |
| --- | --- | --- | --- | --- |
| **Control** | 135 | 54.9 ±11.8 | 82:53 | N/A |
| **Early PD** | 73 | 62. 1±7.0 | 39:34 | 10.5 ±3.4 |
| **Mid PD** | 98 | 64.5 ±8.0 | 52:46 | 21.9 ±4.1 |
| **Late PD** | 34 | 65.5 ±6.8 | 14:20 | 40.0±8.1 |

**Table S3** Primer sequences

| Gene name | Forward (5’ to 3’) | Reverse (5’ to 3’) |
| --- | --- | --- |
| α-synuclein | GCAGAGGCAGCTGGAAAGACAA | CCTCTGAAGGCATTTCATAAGCCTCACT |
| CHCHD2 | TGAGTCCAGGCTCTGCCCATTA | CAGCGAAGACAAACTGCGAAAC |
| GAPDH | TGACGTGCCGCCTGGAGAAA | AGTGTAGCCCAAGATGCCCTTCAG |
| P300 | TGCCAACCCTAATCCACAGC | TGCCTGCAGAGGATTCATGT |
| CHCHD2 (Chip) | TGCCAATCAATGGGCTCAGGTC | GGTTTCAGACTGTGATTTAGGGA |

**Table S4.** Demographics of post-mortem control and PD patients.

| Group | Brain bank number | Sex | Age | Clinical diagnosis | Neurological examination | Braak stages | Death time |
| --- | --- | --- | --- | --- | --- | --- | --- |
| Control | 2018CBB036 | Male | 65 | Advanced liver cancer, multiple organ failure | No obvious abnormalities | 0 | 2018.11 |
|  | 2019CBB050 | Male | 70 | Pulmonary infection, heart failure, acute leukemia, severe anemia | No obvious abnormalities | 0 | 2019.11 |
|  | 2019CBB009 | Male | 72 | Severe pneumonia (invasive pulmonary mycosis) ;Type I respiratory failure; | No obvious abnormalities | 0 | 2019.04 |
| PD | 2015-CBB24 | Male | 79 | Cerebral infarction, Parkinson's disease, lung infection, septic shock | Parkinson's disease | 3 | 2015.10 |
|  | 2016CBB0027 | Male | 91 | Coronary heart disease | Parkinson's disease | 3 | 2016.10 |
|  | 2018CBB038 | Male | 66 | Septic shock, lung infection, asphyxia | Parkinson's disease | 3 | 2018.12 |
